# Supine posture changes lung volumes and increases ventilation heterogeneity in cystic fibrosis

**DOI:** 10.1371/journal.pone.0188275

**Published:** 2017-11-27

**Authors:** Laurie J. Smith, Kenneth A. Macleod, Guilhem J. Collier, Felix C. Horn, Helen Sheridan, Ina Aldag, Chris J. Taylor, Steve Cunningham, Jim M. Wild, Alex Horsley

**Affiliations:** 1 POLARIS, Academic Radiology, University of Sheffield, Sheffield, United Kingdom; 2 Sheffield Children’s Hospital NHS Foundation Trust, Sheffield, United Kingdom; 3 Royal Hospital for Sick Children, Edinburgh, United Kingdom; 4 Respiratory Research Group, Division of Infection, Immunity & Respiratory Medicine, University of Manchester, Manchester, United Kingdom; University Children`s Hospital Zurich, SWITZERLAND

## Abstract

**Introduction:**

Lung Clearance Index (LCI) is recognised as an early marker of cystic fibrosis (CF) lung disease. The effect of posture on LCI however is important when considering longitudinal measurements from infancy and when comparing LCI to imaging studies.

**Methods:**

35 children with CF and 28 healthy controls (HC) were assessed. Multiple breath washout (MBW) was performed both sitting and supine in triplicate and analysed for LCI, S_cond_, S_acin_, and lung volumes. These values were also corrected for the Fowler dead-space to create ‘alveolar’ indices.

**Results:**

From sitting to supine there was a significant increase in LCI and a significant decrease in FRC for both CF and HC (p<0.01). LCI, when adjusted to estimate ‘alveolar’ LCI (LCI_alv_), increased the magnitude of change with posture for both LCI_alv_ and FRC_alv_ in both groups, with a greater effect of change in lung volume in HC compared with children with CF. The % change in LCI_alv_ for all subjects correlated significantly with lung volume % changes, most notably tidal volume/functional residual capacity (Vt_alv_/FRC_alv_ (r = 0.54,p<0.001)).

**Conclusion:**

There is a significant increase in LCI from sitting to supine, which we believe to be in part due to changes in lung volume and also increasing ventilation heterogeneity related to posture. This may have implications in longitudinal measurements from infancy to older childhood and for studies comparing supine imaging methods to LCI.

## Introduction

Multiple breath washout (MBW) is recognised as an important clinical and research method in cystic fibrosis (CF) [1]. Indices derived from MBW reflect the degree of ventilation heterogeneity within the lungs. The lung clearance index (LCI) is thought to reflect overall ventilation heterogeneity, whereas the phase III slope indices S_cond_ and S_acin_ are proposed indices of heterogeneity in the washout signal from the convection and diffusion-convection dependent airways respectively [2]. LCI in particular has been extensively investigated and is an important measure of early lung disease in CF [3, 4] and is well suited to a paediatric age group with relatively mild lung disease.

The effect of posture on LCI in children with CF has recently been assessed [5]. This study compared CF children with mild disease to healthy controls (HC) and showed that LCI in the supine posture was elevated when compared to sitting and that supine LCI correlated better than sitting LCI with metrics derived from high resolution computed tomography (HRCT). The authors also reported that the magnitude of the increase in LCI was greater in CF children than HC, despite similar reductions in FRC. This postural effect is also important when assessing longitudinal changes in LCI, with infant MBW performed supine whilst older children tend to perform the measurements sitting. The effect of this change in posture is therefore important for the interpretation of changes in LCI.

One major effect of the supine posture is the impact that this has on lung volumes. In particular, a reduction in FRC when lying supine compared to an upright posture is widely reported [6].

LCI is calculated using the FRC and several authors have shown that LCI can be affected by large changes in lung volumes [7–9]. Understanding the effect of posture on ventilation heterogeneity may allow more accurate comparisons with imaging and relate structural abnormalities to lung physiology and will aid in the interpretation of longitudinal studies in young children.

Further to the work of Ramsey et al [5], the aim of this study therefore was to compare LCI and the phase III slope parameters S_cond_ and S_acin_, sitting and supine, in children with a range of CF lung disease as well as in HC, and to measure the extent to which changing lung volume affects the change in LCI. We hypothesised that supine posture would firstly reduce lung volume (FRC) with a resultant effect on gas mixing, but also that there would be an additional effect of posture on ventilation heterogeneity within the lungs.

## Methods

This was an observational study performed at two specialist UK paediatric centres: Sheffield Children’s Hospital (SCH) and Royal Hospital for Sick Children, Edinburgh (RHSC). This study was approved by the National Research and Ethics Committee (SCH—REC 12/YH/0343. RHSC—Lothian Research Ethics Committee (08/S1101/290)) and parents/guardians provided written informed consent.

Subjects with CF had a clinical diagnosis confirmed via sweat testing and/or genetic testing and were clinically stable, without exacerbation for at least 4 weeks prior to assessment. HC were without chronic respiratory illness, neuromuscular weakness or bone disease likely to impair respiration and with no congenital cardiac disease requiring medication. HC also had no history of acute respiratory illness in the 4 weeks prior to testing. Children were aged 5–16 years old.

LCI values from SCH CF (n = 19) and HC (n = 10) children have previously been reported [10]. This previous work reported the sensitivity of LCI to detect lung disease in comparison to helium-3 MRI, spirometry and CT. This report did not compare the sitting LCI to that performed in the supine posture. The current study also contains additional analyses to explore the causes of the changes in LCI on lying supine.

All subjects performed MBW using a modified open-circuit Innocor device with (0.2%) sulfa-hexaflouride (SF_6_) as the tracer gas for wash-in and room air for washout [11]. MBW was measured in triplicate without randomisation with regards the order of sitting and supine measurements. MBW was firstly performed seated and then supine, followed by spirometry. Spirometry was performed according to international guidelines [12] using a ‘PFT Pro’ (Carefusion, Basingstoke, UK) in the SCH cohort and using an ‘Easyone’ (ndd, Zurich, Switzerland) in the RHSC cohort. The SCH cohort did not pause after the supine posture was adopted before performing MBW, whilst the RHSC cohort waited 30 minutes whilst supine before starting MBW.

The Innocor MBW system was used in both centres as previously described [11]. Each subject breathed tidally through a low dead-space filter and flanged mouthpiece (adapted to ensure minimal dead-space) attached in series to a pneumotachograph (Hans-Rudolph, USA). During the wash-in, an open bias flow circuit was attached to the distal end of the pneumotachograph, supplying 0.2% SF_6_ in air at a flow rate of 10-12lpm from a gas cylinder. Each subject was encouraged to breathe naturally whilst being distracted by watching a DVD until the difference between the inspired and expired concentration of SF_6_ was <0.004%. The subject was then disconnected from the flow-past circuit during exhalation, after which the subject continued tidal breathing until the expired concentration of SF_6_ dropped below 1/40^th^ of the initial concentration (approximately 0.005%) for three consecutive breaths.

MBW data were analysed using custom-built software for the measurements of LCI, S_cond_ and S_acin,_ FRC, tidal volume ((Vt) taken as the average across all washout breaths) and Fowler dead-space (FDS) [13], with the final result being the average value recorded from at least 2 acceptable trials [14]. LCI was calculated as the cumulative expired volume required to ‘wash-out’ the tracer gas to 1/40^th^ of the ‘washed-in’ concentration, divided by the FRC. Phase III slope analysis was automated and checked manually to confirm accurate identification of alveolar slopes. S_acin_ was calculated from the first expiratory breath after the wash-in was complete. S_cond_ was calculated from breaths during 1.5–6 lung turnovers as previously described [14]. Spirometric measures of FEV_1_, FVC and FEV_1_/FVC were expressed as z-scores using recommended equations [15].

### Methods for alveolar indices of MBW

Alveolar indices were calculated as previously described [16]. FDS [17] was calculated from the exhaled CO_2_ curve measured at each breath of the wash-out and the average from all breaths recorded. Alveolar lung volumes were then calculated by subtracting the FDS from the lung volumes to create FRC_alv_ and Vt_alv_. LCI_alv_ was calculated using the formula:

$${LCI}_{alv}=\frac{\left(\mathrm{CEV-}(\mathrm{FDS}\times B)\right)}{\left(\mathrm{FRC-FDS}\right)}$$

Where CEV is the cumulative expired volume required to wash-out SF_6_ to 1/40^th^ of the starting concentration and B is the number of tidal breaths required to do the same.

### Statistical analysis

Comparisons between CF and HC were performed using un-paired t-tests or Mann-Whitney tests after assessing for the normal distribution. Comparisons between sitting and supine measurements made in the same subjects were performed using paired t-tests or the Wilcoxon matched-pairs signed rank test. Correlations were performed using either Pearson or Spearman rank analysis. A p-value of <0.05 was considered statistically significant and presented throughout the manuscript alongside the mean or median difference (MD) and the 95% confidence intervals (CI) where appropriate. All analysis was performed using GraphPad Prism V7 (San Diego, USA). Height and weight were converted into age-appropriate z-scores [18].

## Results

35 children with CF (19 from SCH) and 28 HC (10 from SCH) completed both the sitting and supine MBW assessments. Demographic and baseline lung function data are summarised in Table 1. HC subjects were significantly taller and heavier than CF subjects (MD (95% CI): height z-score = -0.73 (-1.24, -0.22,), p = 0.006. Weight z-score = -0.43 (-0.79, 0.00), p = 0.049)) and non-significant trends of greater age in HC. CF subjects had significantly poorer lung function outcomes than HC, with lower FEV_1_ and FEV_1_/FVC z scores as well as increased sitting LCI and S_cond_. Despite this, in CF subjects the overall mean z-scores for FEV_1_ and FEV_1_/FVC were -0.78 and -0.89 respectively, and therefore within the normal reference range (z-score >-1.64). 8/35 CF subjects had an FEV_1_ z-score <-1.64, and 6/35 had an FEV_1_/FVC z-score <-1.64. In contrast, 17/35 subjects had an LCI (sitting) greater than the upper limit of normal (7.4) [11]. All HCs had normal FEV_1_, FEV_1_/FVC and LCI (sitting).

**Table 1:** Demographics and baseline lung function data for healthy control and cystic fibrosis subjects.

	Healthy Controls	Cystic Fibrosis	Mean or median difference (95% CI)	p value
Number of subjects	28	35		
Sex (M/F)	16/12	16/19		
Age (years)	11.53 (3.39)	10.07 (2.82)	-1.45 (-3.02, 0.11)	0.068
Height (cm)	150.30 (20.27)	137.30 (17.30)	-12.99 (-22.46, -3.52)	0.008
Height z-score	0.59 (0.96)	-0.14 (1.04)	-0.73 (-1.24, -0.22,)	0.006
Weight (kg)	44.54 (18.46)	35.49 (13.68)	-7.15 (-17.3, 0.3)	0.065
Weight z-score	0.57 (0.97)	0.23 (0.76)	-0.43 (-0.79, 0.00)	0.049
FEV_1_ (z-score)	-0.07 (0.86)	-0.78 (1.31)	-0.52 (-1.09, -0.04)	0.033
FEV_1_/FVC (z-score)	-0.09 (0.81)	-0.89 (0.80)	-0.78 (-1.21, -0.33)	<0.001
LCI (sitting)	6.25 (0.41)	7.72 (1.68)	1.04 (0.60, 1.64)	<0.001
LCI (supine)	6.47 (0.40)	8.22 (1.86)	1.1 (0.66, 1.76)	<0.001
FRC (L) (sitting)	1.85 (0.78)	1.25 (0.43)	-0.44 (-0.77, -0.19)	<0.001
S_cond_ (sitting)	0.02 (0.01)	0.05 (0.02)	0.03 (0.02, 0.05)	<0.001
S_acin_ (sitting)	0.10 (0.04)	0.15 (0.09)	0.02 (-0.01, 0.05)	0.087

Values are expressed as mean (SD). Mann-Whitney tests demonstrate whether the two groups significantly differ for each outcome, with the exception of age and height, which were assessed using un-paired t-tests. The mean or median difference, 95% confidence intervals and p-value are presented.

Combined CF and HC demographics compared between each testing centre were not significantly different. CF patients from SCH were however, slightly older than RHSC (mean age = 10.93 vs 9.06 years, p = 0.049) and therefore also taller (mean height = 142.8 vs 130.8cm, p = 0.039). There were no significant differences when comparing the % change in LCI, FRC, CEV, Vt or Vt/FRC between subjects measured from the two different centres. The SCH cohort demonstrated no significant difference or consistent effect between supine MBW trial 1 and trial 3 (p = 0.68).

### Changes with posture

**Fig 1:**
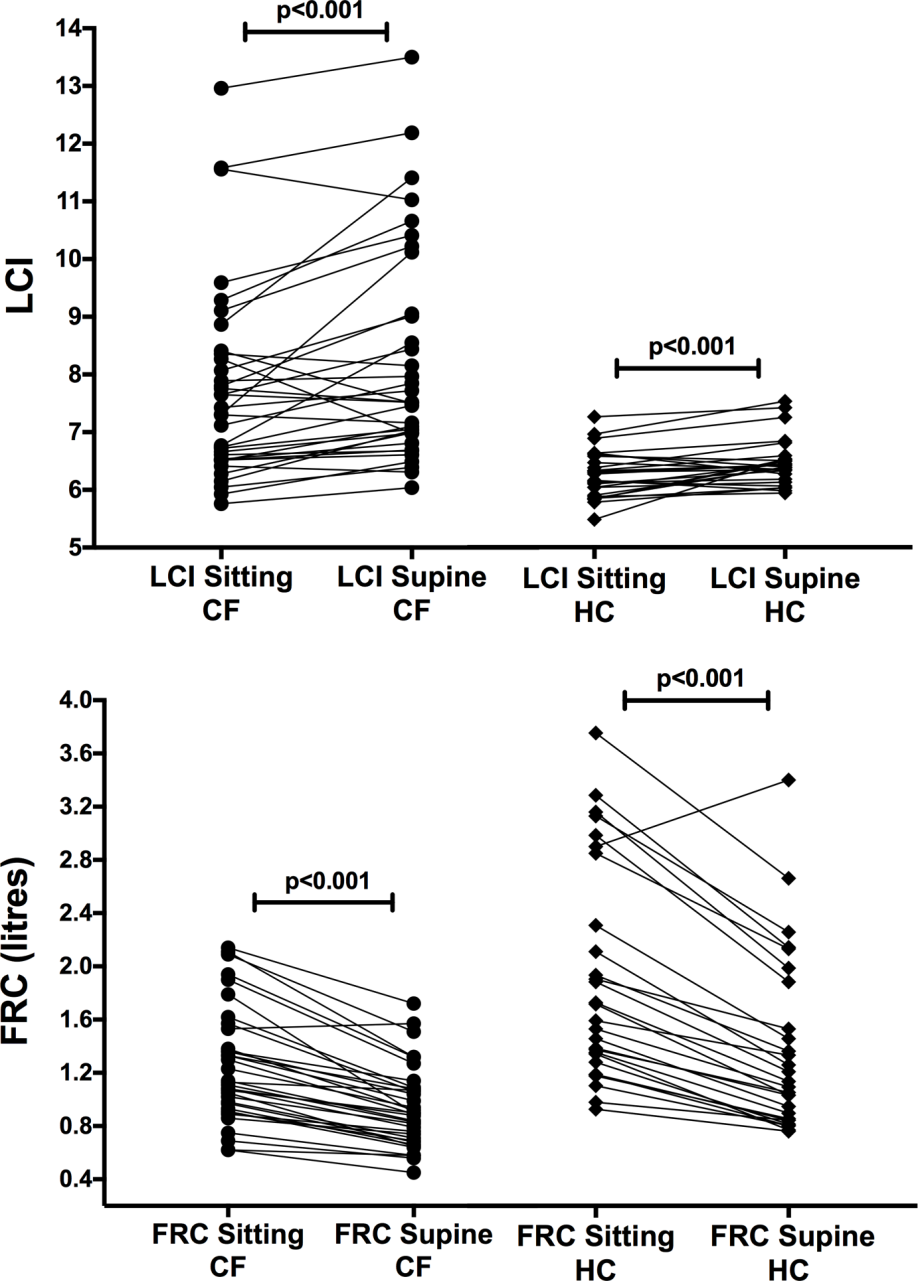
LCI and FRC change from sitting to supine in both cystic fibrosis (CF) and healthy controls (HC). There is a significant increase in LCI from sitting to supine in both CF and HC and a significant decrease in FRC.

There were large and statistically significant reductions in FRC when changing from the sitting to supine posture, in both CF and HC, of 24.40% (95% CI = 20.95, 27.85) and 28.42% (95% CI = 23.92, 32.91) respectively (both p<0.001) (see Table 2, Fig 1). There were small but statistically significant increases in LCI. In HC, mean LCI increased by 3.63% from 6.25 to 6.47 (MD (95% CI) = 0.17 (0.06, 0.36), p<0.001) and in CF LCI increased by 6.69% from 7.72 to 8.22 (MD (95% CI) = 0.34 (0.17, 0.72), p<0.001). There were also large and statistically significant reductions in CEV. In CF, mean CEV decreased by 20.00% (95% CI = 16.00, 24.90) and in HC by 25.40% (95% CI = 21.22, 29.63). Adjusting FRC for height by expressing as a percentage of predicted did not change the pattern of results.

**Table 2:** Sitting to supine comparison of multiple breath washout (MBW) outcomes for both cystic fibrosis (CF) and healthy control (HC) subjects.

	Sitting	Supine	% Change	Mean or median difference (95% CI)	p value
LCI HC	6.25 (0.4)	6.47 (0.4)	3.63 (5.26)	0.17 (0.06, 0.36)	<0.001
LCI CF	7.72 (1.68)	8.22 (1.86)	6.69 (10.41)	0.34 (0.17, 0.72)	<0.001
FRC HC (L)	1.85 (0.78)	1.32 (0.64)	-28.42 (11.59)	-0.47 (-0.66, -0.34)	<0.001
FRC CF (L)	1.25 (0.43)	0.93 (0.30)	-24.40 (10.04)	-0.28 (-0.34, -0.21)	<0.001
Vt HC (L)	0.45 (0.20)	0.46 (0.20)	7.72 (28.01)	0.01 (-0.04, 0.06)	0.682
Vt CF (L)	0.42 (0.15)	0.39 (0.15)	-8.04 (13.46)	-0.03 (-0.07, -0.01)	<0.001
Vt/FRC HC (%)	25.61 (10.50)	37.5 (14.40)	54.5 (44.78)	11.68 (6.75, 15.53)	<0.001
Vt/FRC CF (%)	35.62 (12.92)	42.96 (14.47)	24.00 (26.42)	7.46 (3.86, 10.96)	<0.001
CEV HC (L)	11.54 (4.7)	8.63 (4.04)	-25.43 (10.84)	-2.73 (-3.74, -2.01,)	<0.001
CEV CF (L)	9.79 (4.09)	7.77 (3.41)	-20.43 (12.97)	-1.79 (-2.53, -1.17)	<0.001
FDS HC (L)	0.09 (0.03)	0.09 (0.03)	8.20 (52.37)	-0.01 (-0.01, 0.01)	0.597
FDS CF (L)	0.08 (0.02)	0.08 (0.02)	-8.39 (11.82)	-0.01 (-0.01, 0.00)	<0.001
S_cond_ HC	0.02 (0.01)	0.03 (0.01)	101.80 (135.90)	0.01 (-0.01, 0.02)	0.028
S_cond_ CF	0.05 (0.02)	0.06 (0.03)	29.60 (59.71)	0.01 (-0.01, 0.16)	0.051
S_acin_ HC	0.10 (0.04)	0.09 (0.04)	-11.71 (41.58)	-0.01 (-0.03, 0.01)	0.117
S_acin_ CF	0.15 (0.09)	0.14 (0.10)	6.50 (42.48)	0.01 (-0.02, 0.02)	0.721

Values are expressed as mean (SD). Wilcoxon tests were calculated to assess whether the sitting to supine change was significantly different, with the exception of FDS HC, S_cond_ CF and S_acin_ HC, which were calculated using paired t-tests. The mean or median difference, 95% confidence intervals and p-value are presented.

**Fig 2:**
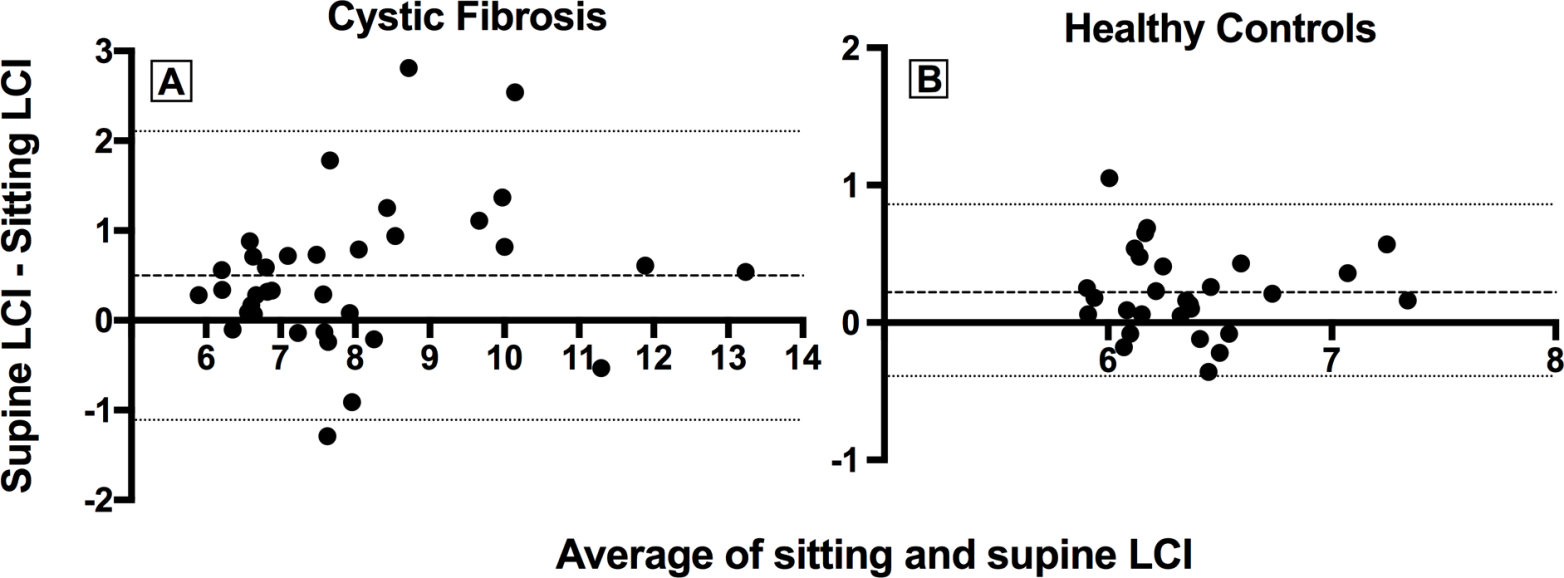
**Bland-Altman analysis of sitting and supine LCI in children with CF (A) and healthy controls (B).** The dashed line indicates the mean difference and the dotted lines indicate the 95% limits of agreement.

Bland-Altman analysis of sitting and supine LCI (Fig 2) demonstrates that the effect of posture on LCI is greater in CF subjects than HC. CF subjects had an increased mean difference (CF = 0.5, HC = 0.22) and wider 95% limits of agreement (2.11, -1.11) when compared to HC (0.83, -0.39).

In CF, supine posture was associated with significant decreases in both Vt (Mean (95% CI) = -8.0% (-12.7, -3.4)) and FDS (Mean (95% CI) = -8.4% (-12.5, -4.3)), whereas in HC neither of these variables showed significant change. In both CF and HC however, the large reductions in FRC led to significant increases in the ratio of Vt/FRC (Mean (95% CI) = 24.0% (14.92, 33.07) and 54.5% (37.2, 71.9) increase respectively).

S_cond_ was low in HC and increased from 0.02 sitting, to 0.03 supine (MD (95% CI) = 0.01 (-0.01, 0.02), p = 0.028). There were no statistically significant changes in other phase III slope indices.

### Analysis of alveolar MBW

Correcting MBW for FDS to produce measures of ventilation heterogeneity independent of changes in physiological dead space did not alter the pattern of changes described above when moving from a sitting to a supine posture. Correcting for FDS increased the magnitude of the postural effect on LCI in both groups (Mean (95% CI) increase of 9.6% (5.5, 13.6) in CF and 8.6% (5.7, 11.6) increase in HC) and the decrease in FRC (Mean (95% CI) = -25.4% (-29.2, -21.7) in CF and -29.4% (-34.2, -24.6) in HC) (Table 3). HC subjects had a significantly larger % change in Vt_alv_/FRC_alv_ than CF but there was no group difference for the % change in LCI_alv_ (Table 4).

**Table 3:** Sitting to supine comparison of ‘alveolar’ multiple breath washout outcomes.

	Sitting	Supine	Mean or median difference (95% CI)	p-value
LCI_alv_ HC	5.16 (0.29)	5.60 (0.43)	0.44 (0.29, 0.59)	<0.001
LCI_alv_ CF	6.65 (1.47)	7.27 (1.66)	0.59 (0.3, 10.73)	<0.001
FRC_alv_ HC (L)	1.77 (0.77)	1.25 (0.63)	-0.44 (-0.69, -0.33)	<0.001
FRC_alv_ CF (L)	1.17 (0.41)	0.86 (0.29)	-0.27 (-0.34, -0.2)	<0.001
Vt_alv_ HC (L)	0.37 (0.17)	0.38 (0.20)	-0.01 (-0.04, 0.05)	>0.99
Vt_alv_ CF (L)	0.34 (0.15)	0.31 (0.14)	-0.03 (-0.05, 0.00)	0.005
Vt_alv_/FRC_alv_ HC (%)	22.64 (10.02)	32.94 (15.34)	7.79 (5.32, 12.04)	<0.001
Vt_alv_/FRC_alv_ CF (%)	30.83 (13.36)	37.78 (15.18)	6.62 (3.36, 9.94)	0.001

Alveolar outcomes were calculated using the method of Haidopoulou et al (2012) [16] which corrects outcomes for the fowler dead-space. Values are expressed as mean (SD). Wilcoxon tests were calculated to determine whether the sitting to supine change was significantly different, with the exception of LCI_alv_ HC, which was calculated using paired t-tests. The mean or median difference, 95% confidence intervals and p-value are presented.

**Table 4:** A comparison of the % change in ‘alveolar’ multiple breath washout outcomes between healthy controls and cystic fibrosis subjects.

% Change	Healthy controls	Cystic fibrosis	Mean or median difference (95% CI)	p-value
LCI_alv_	8.62 (7.45)	9.57 (11.74)	0.38 (-3.76, 5.31	0.844
FRC_alv_	-29.43 (12.15)	-25.43 (10.88)	5.00 (0.19, 9.47)	0.042
Vt_alv_	3.75 (24.53)	-7.95 (15.19)	11.7 (1.56, 21.83)	0.024
Vt_alv_/FRC_alv_	50.63 (44.15)	27.16 (30.69)	23.47 (4.45, 42.49)	0.017

Comparsions made using un-paired t-tests, with the exception of LCI_alv_ and FRC_alv_, which were calculated using Mann-Whitney tests. The mean or median difference, 95% confidence intervals and p-value are presented.

## Correlations

**Fig 3:**
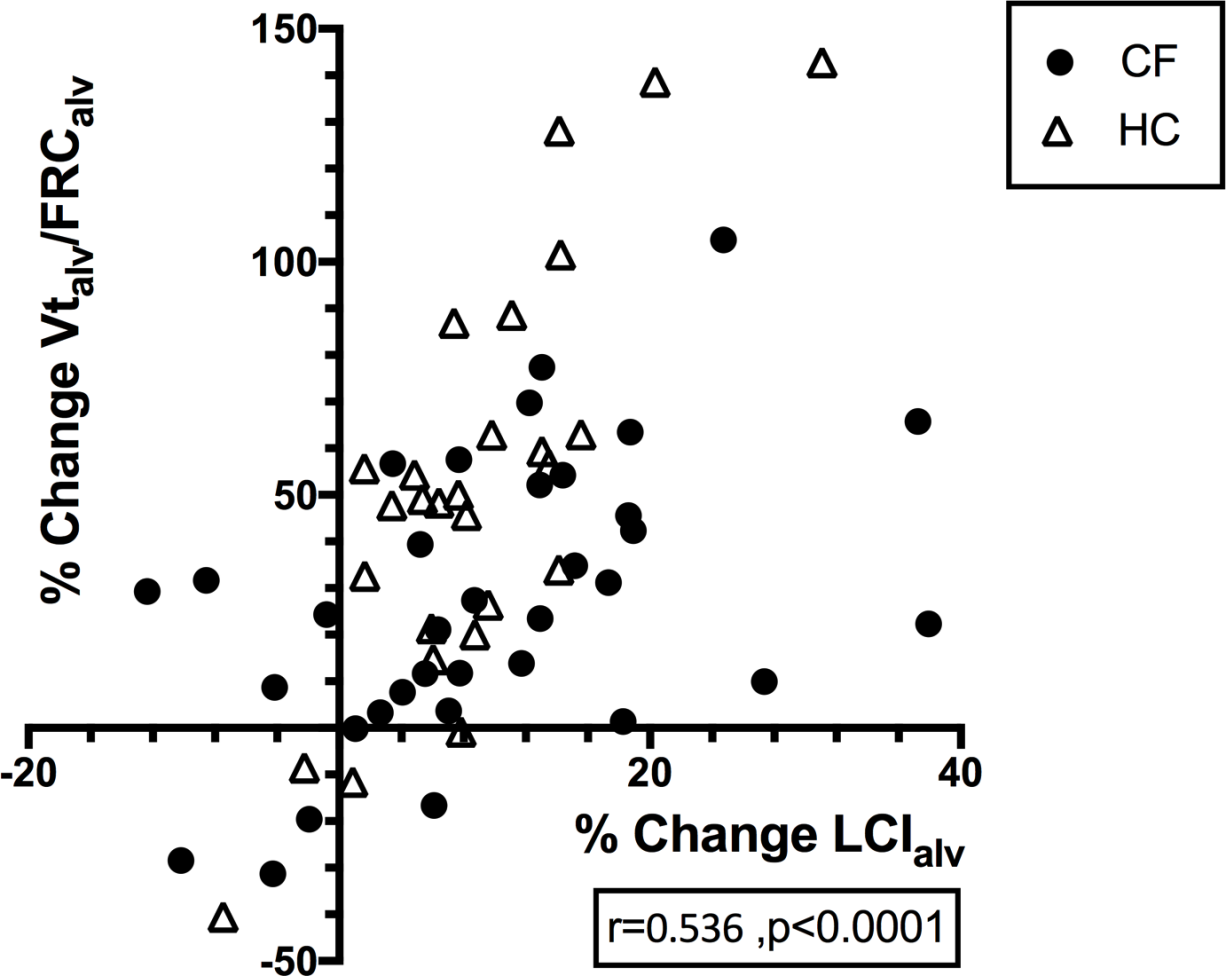
Scatter plot with Spearman correlation of the % change in the ‘alveolar’ Vt_alv_/FRC_alv_ with the % change in ‘alveolar’ LCI_alv_ for both cystic fibrosis (CF) and healthy control (HC) subjects combined. The Spearman correlation suggests a positive relationship between increasing % change Vt_alv_/FRC_alv_ and increasing % change LCI_alv_.

In the combined CF and HC data, the difference in LCI from sitting to supine and the % change in LCI, resulted in no significant correlations with any baseline or % change value. For the combined CF and HC populations, the % change in LCI_alv_ however showed significant correlations with the % change in FRC_alv_ (r = -0.47, p<0.001), % change in Vt_alv_ (r = 0.38, p = 0.002) and the % change in Vt_alv_/FRC_alv_ (r = 0.54, p<0.001, Fig 3). Individually, the % change in Vt_alv_/FRC_alv_ against % change in LCI_alv_ was better correlated in HC (r = 0.71, p<0.001) than in CF (r = 0.50, p = 0.002).

## Discussion

These results demonstrate that subjects with CF and predominantly mild lung disease increase LCI when changing posture from sitting to supine, with an associated significant reduction in FRC. This study adds to previously reported data by interrogating possible causes for the increase seen in LCI including the effects of dead-space and the change in different lung volumes due to posture [5]. When assessing lung volumes measured during MBW, in addition to a reduction in FRC, we also demonstrate in CF subjects that adopting the supine posture resulted in a decrease in Vt and FDS. All subjects had a significant increase in the ratio of Vt/FRC and decrease in CEV.

The increase in LCI with change in position suggests this change of posture increases ventilation heterogeneity in both CF and HC. The relative (%) change however did not correlate with other measured baseline parameters, including other markers of disease severity. There were inconsistent changes in S_cond_ and S_acin_ when adopting the supine posture, we are therefore unable to determine whether the increasing heterogeneity was specifically related to changes in conductive or acinar airway heterogeneity.

### Alveolar LCI

To assess whether increased LCI was due to increasing ventilation heterogeneity and not due to changes in physiological dead-space, the method of Haidopoulou [16] was used to calculate the ‘alveolar’ LCI (LCI_alv_). This involves subtracting the Fowler dead-space from the lung volume components and reduces LCI. The observation that LCI_alv_ has a greater increase than LCI from sitting to supine confirms that increased supine LCI is not simply a mathematical consequence of reducing FRC. Correcting for the dead-space also changed the relationship between the outcomes, such that the % change in LCI_alv_ became significantly correlated with the mean Vt_alv_, FRC_alv_ and therefore the Vt_alv_/FRC_alv_ ratio. The correlation between the % change in Vt_alv_/FRC_alv_ and LCI_alv_ was stronger in HC than CF suggesting that the change in lung volumes, from sitting to supine, is a more important factor in the change in LCI in subjects without airways disease. In patients with CF, even in those with mild disease, there is a less predictable effect of posture on the distribution of ventilation.

Consistent with this, CF subjects also had a poorer agreement between LCI values measured sitting and supine than HC, again suggesting that CF lungs have a more unpredictable gas mixing response to the supine posture than HC.

### Relevance

Imaging studies performed supine, such as MRI [10, 19–21] and CT [22] are increasingly used to assess the earliest changes in the CF lung. MBW performed while seated is also being used to provide regional insight to the structural and physiological changes measured in MBW [23]. In addition, longitudinal studies in young children with CF often compare measurements performed in infancy while supine, with repeated measurements performed seated in later childhood. Age related change in posture when measuring LCI should also be taken into consideration when interpreting longitudinal studies. The results of this study suggest; (i) that the supine posture changes LCI, (ii) that the amount it changes by is not predictable in an individual and (iii) that there is likely a true increase in ventilation heterogeneity [5]. Due to the inherent nature of how LCI is calculated, a change in FRC and Vt/FRC will lead to a change in LCI [9]. Our results suggest that the increase in LCI seen in CF is related to changes in ventilation heterogeneity. Therefore, subject posture should be taken into consideration when making direct physiological comparisons between MBW and imaging outcomes[5] and when assessing longitudinal measurements.

Our findings are predominantly aligned with those previously reported, except we found no significant difference in the amount of change in LCI, from sitting to supine, between CF and HC [5]. The reason for this difference is not clear, though it may relate to the different MBW gases used;. SF_6_ wash-in and washout as reported here and a Nitrogen (N_2_) washout methodology previously reported[5]. LCI derived from SF_6_ measurement is likely to differ from N_2_ LCI methodology[14], due to the differing gas properties and also possible affects of N_2_ resident in the lung and excreted from alveolar tissue. There are also discrepancies with regards FRC measurements from differing N_2_ washout systems, [24, 25] which may also have an impact on postural measurements. The CF cohort reported has a younger mean age (by approximately 2 years), with a lower mean sitting LCI than previously reported [5], however the HC cohorts have a similar disparity in sitting LCI in both studies. This makes it difficult to determine whether there is therefore a true difference in disease severity of the two CF cohorts or whether the difference is due to differing MBW methodologies.

Imaging studies demonstrate that ventilation gradients are present in healthy lungs, that are liable to change when lying supine [26] or in microgravity [27]. Supine changes in LCI may therefore be due to altered gravitational forces. Such changes appear poorly predictable in CF lungs where there is greater variation in regional lung compliance and airway calibre than the healthy lung [28]. This may also explain why children with asthma [29] and adults with chronic heart failure [30] demonstrate no change in LCI when lying supine despite similarly reduced FRC. In this study we have only assessed patients with predominantly mild CF, but it is possible that the effect may be more profound or more unpredictable in those with more severe disease.

Mucus plugging and bronchiectasis are common features in CF lungs [28]. It is possible that when lying supine, where the distribution of ventilation causes increased inter-regional ventilation heterogeneity [31], that any peripheral areas of bronchiectasis and mucus plugging receive a greater distribution of ventilation compared to an upright posture, causing an increase in ventilation heterogeneity and therefore LCI.

### Study limitations

Data collection for this study was carried out at two separate centres, with the data combined. Subjects from RHSC waited for 30 minutes in the supine posture before measuring MBW whilst SCH subjects only waited the time it took to position the subject and equipment (5-10mins). When we compared data from the two centres however there was no significant difference in the % change from sitting to supine for any of the outcomes studied. We have assumed that this difference in supine time was not important, as the SCH cohort demonstrated no significant tendency to increasing LCI values with repeated measurements in the supine position. Another limitation of this study is that we predominantly studied CF children with mild lung disease, therefore the effects of posture change in more severe disease and in adult patients with CF is not known. We also did not randomise the order in which the subjects performed either sitting or supine MBW. Ideally the order would have been randomly assigned, however for both centres the MBW measurements were made as part of larger separate studies where sitting MBW was the primary outcome [10]. We can therefore not fully assess whether there was a learning effect from repeated MBW measurements.

## Conclusion

We have demonstrated that children with CF and HC have an increase in LCI and a decrease in FRC when lying supine that is not simply due to changes in lung volumes. The change in LCI appears partly driven by an increase in Vt/FRC when supine and in HC may be the main factor contributing to this change in LCI. However, in CF subjects the changes in lung volumes can only explain a proportion of the change in LCI, which is also caused by true increases in ventilation heterogeneity. The effect of posture is hard to predict on an individual basis and care should be taken when correlating supine imaging measurements with physiology assessments performed seated.

## References

[1] EMA. European Medicines Agency: Report of the workshop on endpoints for cystic fibrosis clinical trials. London: 2012.

[2] Verbanck S, Paiva M. Gas mixing in the airways and airspaces. Comprehensive Physiology. 2011;1(2):809–34. Epub 2011/04/01. doi: 10.1002/cphy.c100018 23737204 10.1002/cphy.c100018

[3] Horsley A, Siddiqui S. Putting lung function and physiology into perspective: cystic fibrosis in adults. Respirology. 2015;20(1):33–45. Epub 2014/09/16. doi: 10.1111/resp.12382 25219816 10.1111/resp.12382

[4] Aurora P, Gustafsson P, Bush A, Lindblad A, Oliver C, Wallis CE, et al Multiple breath inert gas washout as a measure of ventilation distribution in children with cystic fibrosis. Thorax. 2004;59(12):1068–73. doi: 10.1136/thx.2004.022590 15563707 10.1136/thx.2004.022590 PMC1746917

[5] Ramsey KA, McGirr C, Stick SM, Hall GL, Simpson SJ, Arest CF. Effect of posture on lung ventilation distribution and associations with structure in children with cystic fibrosis. Journal of cystic fibrosis: official journal of the European Cystic Fibrosis Society. 2017 doi: 10.1016/j.jcf.2017.01.013 28188011 10.1016/j.jcf.2017.01.013

[6] West JB. Respiratory physiology: the essentials Baltimore, MD London: Baltimore, MD London: Lippincott Williams & Wilkins; 2012.

[7] Lum S, Stocks J, Stanojevic S, Wade A, Robinson P, Gustafsson P, et al Age and height dependence of lung clearance index and functional residual capacity. European Respiratory Journal. 2013;41(6):1371–7. doi: 10.1183/09031936.00005512 23143552 10.1183/09031936.00005512

[8] Schmalisch G, Wilitzki S, Buhrer C, Fischer HS. The lung clearance index in young infants: impact of tidal volume and dead space. Physiol Meas. 2015;36(7):1601–13. Epub 2015/06/19. doi: 10.1088/0967-3334/36/7/1601 26086894 10.1088/0967-3334/36/7/1601

[9] Yammine S, Singer F, Gustafsson P, Latzin P. Impact of different breathing protocols on multiple-breath washout outcomes in children. Journal of Cystic Fibrosis. 2014;13(2):190–7. http://dx.doi.org/10.1016/j.jcf.2013.08.010 24075581 10.1016/j.jcf.2013.08.010

[10] Marshall H, Horsley A, Taylor CJ, Smith L, Hughes D, Horn FC, et al Detection of early subclinical lung disease in children with cystic fibrosis by lung ventilation imaging with hyperpolarised gas MRI. Thorax. 2017;72(8):760–2. doi: 10.1136/thoraxjnl-2016-208948 28265032 10.1136/thoraxjnl-2016-208948

[11] Horsley AR, Gustafsson PM, Macleod KA, Saunders C, Greening AP, Porteous DJ, et al Lung clearance index is a sensitive, repeatable and practical measure of airways disease in adults with cystic fibrosis. Thorax. 2008;63(2):135–40. Epub 2007/08/07. doi: 10.1136/thx.2007.082628 17675315 10.1136/thx.2007.082628

[12] Miller MR, Hankinson J, Brusasco V, Burgos F, Casaburi R, Coates A, et al Standardisation of spirometry. European Respiratory Journal. 2005;26(2):319–38. doi: 10.1183/09031936.05.00034805 16055882 10.1183/09031936.05.00034805

[13] Hannon D, Bradley JM, Bradbury I, Bell N, Elborn JS, O'Neill K. Shortened Lung Clearance Index is a repeatable and sensitive test in children and adults with cystic fibrosis. BMJ open respiratory research. 2014;1(1):e000031 Epub 2014/12/06. doi: 10.1136/bmjresp-2014-000031 25478180 10.1136/bmjresp-2014-000031 PMC4212720

[14] Robinson PD, Latzin P, Verbanck S, Hall GL, Horsley A, Gappa M, et al Consensus statement for inert gas washout measurement using multiple- and single- breath tests. The European respiratory journal. 2013;41(3):507–22. doi: 10.1183/09031936.00069712 23397305 10.1183/09031936.00069712

[15] Quanjer PH, Stanojevic S, Cole TJ, Baur X, Hall GL, Culver BH, et al Multi-ethnic reference values for spirometry for the 3-95-yr age range: the global lung function 2012 equations. European Respiratory Journal. 2012;40(6):1324–43. doi: 10.1183/09031936.00080312 22743675 10.1183/09031936.00080312 PMC3786581

[16] Haidopoulou K, Lum S, Turcu S, Guinard C, Aurora P, Stocks J, et al Alveolar LCI vs. standard LCI in detecting early CF lung disease. Respiratory physiology & neurobiology. 2012;180(2–3):247–51. Epub 2011/12/17. doi: 10.1016/j.resp.2011.11.016 22172773 10.1016/j.resp.2011.11.016

[17] Fowler WS. Lung function studies; the respiratory dead space. Am J Physiol. 1948;154(3):405–16. Epub 1948/09/01. 18101134 10.1152/ajplegacy.1948.154.3.405

[18] Cole TJ, Freeman JV, Preece MA. British 1990 growth reference centiles for weight, height, body mass index and head circumference fitted by maximum penalized likelihood. Stat Med. 1998;17(4):407–29. 9496720

[19] Stahl M, Wielputz MO, Graeber SY, Joachim C, Sommerburg O, Kauczor HU, et al Comparison of Lung Clearance Index and Magnetic Resonance Imaging for Assessment of Lung Disease in Children With Cystic Fibrosis. American journal of respiratory and critical care medicine. 2016 Epub 2016/08/31. doi: 10.1164/rccm.201604-0893OC 27575911 10.1164/rccm.201604-0893OC

[20] Svenningsen S, Nair P, Guo F, McCormack DG, Parraga G. Is ventilation heterogeneity related to asthma control? The European respiratory journal. 2016;48(2):370–9. Epub 2016/05/14. doi: 10.1183/13993003.00393-2016 27174885 10.1183/13993003.00393-2016

[21] Theilmann RJ, Darquenne C, Elliott AR, Bailey BA, Conrad DJ. Characterizing Lung Disease in Cystic Fibrosis with Magnetic Resonance Imaging and Airway Physiology. PLoS One. 2016;11(6):e0157177 doi: 10.1371/journal.pone.0157177 27337056 10.1371/journal.pone.0157177 PMC4919047

[22] Ramsey KA, Rosenow T, Turkovic L, Skoric B, Banton G, Adams A-M, et al Lung Clearance Index and Structural Lung Disease on Computed Tomography in Early Cystic Fibrosis. Am J Respir Crit Care Med. 2015;193(1):60–7. doi: 10.1164/rccm.201507-1409OC 26359952 10.1164/rccm.201507-1409OC

[23] Horsley A, Wild JM. Ventilation heterogeneity and the benefits and challenges of multiple breath washout testing in patients with cystic fibrosis. Paediatr Respir Rev. 2015;16 Suppl 1:15–8. doi: 10.1016/j.prrv.2015.07.010 26420586 10.1016/j.prrv.2015.07.010

[24] Poncin W, Singer F, Aubriot AS, Lebecque P. Agreement between multiple-breath nitrogen washout systems in children and adults. Journal of cystic fibrosis: official journal of the European Cystic Fibrosis Society. 2016 Epub 2016/12/07. doi: 10.1016/j.jcf.2016.11.004 27919570 10.1016/j.jcf.2016.11.004

[25] Raaijmakers L, Jensen R, Stanojevic S, Ratjen F. Validation of multiple breath washout devices. Journal of Cystic Fibrosis. 2017 https://doi.org/10.1016/j.jcf.2017.01.011 10.1016/j.jcf.2017.01.011 28185888

[26] Horn FC, Deppe MH, Marshall H, Parra-Robles J, Wild JM. Quantification of regional fractional ventilation in human subjects by measurement of hyperpolarized 3He washout with 2D and 3D MRI. Journal of applied physiology (Bethesda, Md: 1985). 2014;116(2):129–39. Epub 2013/12/07. doi: 10.1152/japplphysiol.00378.2013 24311749 10.1152/japplphysiol.00378.2013

[27] Prisk GK, Guy HJ, Elliott AR, Paiva M, West JB. Ventilatory inhomogeneity determined from multiple-breath washouts during sustained microgravity on Spacelab SLS-1. Journal of applied physiology (Bethesda, Md: 1985). 1995;78(2):597–607.

[28] de Jong PA, Lindblad A, Rubin L, Hop WC, de Jongste JC, Brink M, et al Progression of lung disease on computed tomography and pulmonary function tests in children and adults with cystic fibrosis. Thorax. 2006;61(1):80–5. Epub 2005/10/26. doi: 10.1136/thx.2005.045146 16244089 10.1136/thx.2005.045146 PMC2080716

[29] Gustafsson PM. Pulmonary gas trapping increases in asthmatic children and adolescents in the supine position. Pediatric pulmonology. 2003;36(1):34–42. Epub 2003/05/29. doi: 10.1002/ppul.10310 12772221 10.1002/ppul.10310

[30] Kee K, Stuart-Andrews C, Nilsen K, Wrobel JP, Thompson BR, Naughton MT. Ventilation heterogeneity is increased in patients with chronic heart failure. Physiological reports. 2015;3(10). Epub 2015/10/24. doi: 10.14814/phy2.12590 26493954 10.14814/phy2.12590 PMC4632958

[31] Gronkvist M, Bergsten E, Gustafsson PM. Effects of body posture and tidal volume on inter- and intraregional ventilation distribution in healthy men. Journal of applied physiology (Bethesda, Md: 1985). 2002;92(2):634–42. Epub 2002/01/18. doi: 10.1152/japplphysiol.00161.2001 11796675 10.1152/japplphysiol.00161.2001

